# Comparison of the Mechanical Properties and Microstructures of QB2.0 and C17200 Alloys

**DOI:** 10.3390/ma15072570

**Published:** 2022-03-31

**Authors:** Zheng Wang, Jiang Li, Yi Zhang, Chuanming Lv, Ting Li, Jiaqi Zhang, Songxiao Hui, Lijun Peng, Guojie Huang, Haofeng Xie, Xujun Mi

**Affiliations:** 1 State Key Laboratory of Nonferrous Metals and Processes, GRIMAT Group Co., Ltd., Beijing 100088, China; wz6252@163.com (Z.W.); huangguojie@grinm.com (G.H.); xiehaofeng@grinm.com (H.X.); mxj@grinm.com (X.M.); 2 China Academy of Space Technology, Beijing 100094, China; lijiangjyo@163.com (J.L.); zy_pb@163.com (Y.Z.); lvchuanming1987@163.com (C.L.); lt850819@163.com (T.L.); 13772057232@163.com (J.Z.); 3 GRIMAT Engineering Institute Co., Ltd., Beijing 101407, China; 4 General Research Institute for Nonferrous Metals, Beijing 100088, China

**Keywords:** beryllium bronze, tensile strength, microstructure, strengthening mechanisms

## Abstract

As it is known, beryllium bronze, an important copper alloy, is widely used in the field of aerospace. Since the performance of domestic and imported beryllium bronze alloys have obvious differences, domestic beryllium bronze QBe2.0 and imported C17200 alloy were adopted, and the hardness and tensile properties of imported and domestic beryllium bronze alloys in the peak aging state were compared and analyzed. In addition, the microstructure morphologies of the C17200 alloy and QBe2.0 alloy were analyzed by SEM, EBSD, and TEM. This study adopted a data-driven exploration approach to elaborate the differences between C17200 and QBe2.0 alloy. After aging at 300 °C for 2 h (peak aging), the tensile strengths of the C17200 alloy and QBe2.0 alloy were 1357 MPa and 1309 MPa, the yield strengths were 1195 MPa and 1188 MPa, and the elongations were 5.5% and 4.0%, respectively. In the peak-aged state, the grain size, uniformity, small angle grain boundary, and twin density of the C17200 alloy were much better than those of the QBe2.0 alloy, which led to more significant grain refinement and twin strengthening effects. A large amount of γ’ phase, γ phase, and β phase were precipitated in both alloys, but the precipitation density of the γ’ strengthening phase in the C17200 alloy was much greater than that of the QBe2.0 alloy. The C17200 alloy exhibited better mechanical properties under the combined effects of the various strengthening mechanisms, which provided a guideline for the subsequent improvement of domestic alloys and laid a solid foundation for the development of new copper alloys.

## 1. Introduction

Beryllium bronze is an alloy with good overall mechanical, physical, and chemical properties that exhibits high strength, elasticity, wear resistance, fatigue resistance, and corrosion resistance after quenching and aging treatment [1]. Moreover, beryllium bronze also possesses high electrical conductivity, high thermal conductivity, good cold resistance, and nonmagnetic properties [2,3]. Considering that beryllium bronze exhibits higher strength and good overall performance than general bronze and brass alloys, it can meet a series of requirements for the design of resilient contacts of electrical connectors, so it has become the primary material used for making resilient contacts for highly reliable electrical connectors, as well as an important raw material for aerospace applications [4,5].

At present, according to the military standard, the production in the aviation and aerospace field of high reliability elastic military electrical connector contact parts are widely manufactured using QBe2.0 or imported C17200 alloys with age strengthening treatment. However, the mechanical properties of the imported C17200 alloy are significantly better than those of the domestic QBe2.0 alloy [6,7]. Therefore, it is necessary to improve the mechanical properties of the QBe2.0 alloy. As a typical precipitation-reinforced copper alloy, the mechanical properties of beryllium bronzes are closely related to their alloy compositions, processing conditions and heat treatment, and heat treatment is the key influencing factor [8]. Beryllium bronze has good processing performance after heat treatment, and can be processed and formed in any way such as rolling, extrusion, etc., and its elastic limit and relaxation stability are very high [9]. At an aging temperature below 320 °C, the precipitation strengthening phase in the QBe2.0 alloy cannot be fully precipitated, the grain boundaries are coarsened, and there are slight precipitation lines in the grains. As the aging temperature continues to rise, the γ-phase material gathers and grows rapidly, especially near the grain boundaries; thus, the mechanical properties will be reduced [10,11,12]. In addition, the half-aging process after solution treatment of beryllium bronze was studied, and it is found that the machinability decreased, which affected the machining accuracy [13]. At the same time, beryllium bronze can reduce the distortion of parts by pre-aging, sub-aging, and multiple aging, different aging processes have a great effect on the properties of the alloy. The aging temperature should be selected according to the design requirements of the product, and an appropriate aging process should be formulated [14,15]. In summary, although researchers have analyzed the heat treatment system, mechanical properties, and microstructure of the QBe2.0 alloy, the specific causes of the different mechanical properties and the influencing mechanisms of domestic and imported beryllium bronze alloys are still not clear. Therefore, this study selected domestic beryllium bronze QBe2.0 alloy and imported beryllium bronze C17200 alloy to compare and analyze the hardness and tensile properties of the imported and domestic beryllium bronze alloys in different states, which is expected to have a significant positive influence on the development of QBe2.0 beryllium bronze.

## 2. Materials and Methods

C17200 (imported) and QBe2.0 (domestic) alloys were selected as the materials for this experimental study, and their alloy compositions are shown in Table 1. The initial state of QBe2.0 alloy is Y_2_ state and that of C17200 alloy is 1/2H state, and they are both subjected to 21% cold deformation after solution treatment. Then, the initial state alloys were annealed in a chamber annealing furnace for 2 h at both 280 °C and 300 °C, to select the optimal heat treatment regime. The Vickers hardness was conducted on a WILSON VH1150 test instrument (Chicago, IL, USA) with an indentation load of 5 kg and a holding time of 15 s. Tensile specimens were measured on an MTS-WD 3100 tensile test machine (Eden Prairie, MN, USA) with a constant strain rate of 10^−3^ s^−1^. The microstructures of the alloys in the peak-aged states were observed using a Zeiss Axiovert 200 MAT metallurgical microscope (Zeiss, Jena, Germany). A JEOL JSM 7001F field emission scanning electron microscope (JEOL, Tokyo, Japan) was used to observe the fracture morphologies of the samples, and its EBSD probe was used to analyze the grain organization, twinning, and grain boundaries. A JEOL JEM-2010 high-resolution electron microscope (Hillsboro, OR, USA) was also used to observe and analyze the precipitation phases of the two alloys with an operating voltage of 200 KV.

## 3. Results and Discussion

### 3.1. Mechanical Properties

**Figure 1:**
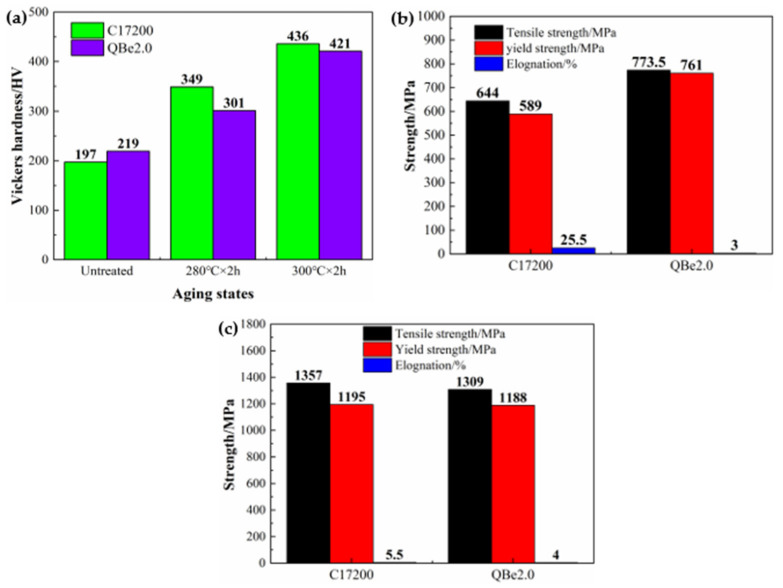
Comparison of the mechanical properties of the alloys at the different conditions. (**a**) Vickers hardness; (**b**) tensile properties of untreated alloys at room temperature; (**c**) tensile properties of peak-aged alloys at room temperature.

Figure 1 compares the mechanical properties of alloys under varying aging conditions, and Figure 1a illustrates the Vickers hardness of the tested alloys at different aging conditions. For instance, the hardness of C17200 at the initial state was higher than that of QBe2.0, which were 219 HV and 197 HV, respectively. After aging treatment, the hardness values of the alloys were greatly improved, and the hardness values after aging for 2 h at 300 °C were obviously higher than those aged at 280 °C. It is worth noting that the hardness values of C17200 were higher than that of QBe2.0, reaching 436 HV and 421 HV at 300 °C, respectively.

Figure 1b,c show the room temperature mechanical properties of the alloys in the initial and peak aging. Based on these two figures, it is found that the yield and tensile strengths are positively related to the hardness. The tensile and yield strengths of C17200 in the initial state are much lower than those of QBe2.0, with tensile strengths of 644 MPa and 774 MPa and yield strengths of 589 MPa and 761 MPa, respectively. The elongation is negatively correlated with the strength of the alloys, which is 25.5% and 3%, respectively. At the peak aging state, the tensile and yield strengths of the alloys were significantly enhanced. The tensile strength exceeds 1300 MPa, and the yield strength exceeds 1100 MPa. Among them, the strengths of C17200 are higher than those of QBe2.0, which are 1357 MPa and 1195 MPa and 1309 MPa and 1188 MPa, respectively. Moreover, the elongation of C17200 is significantly reduced, while QBe2.0 is slightly increased with the result of these two alloys reaching the same level.

**Figure 2:**
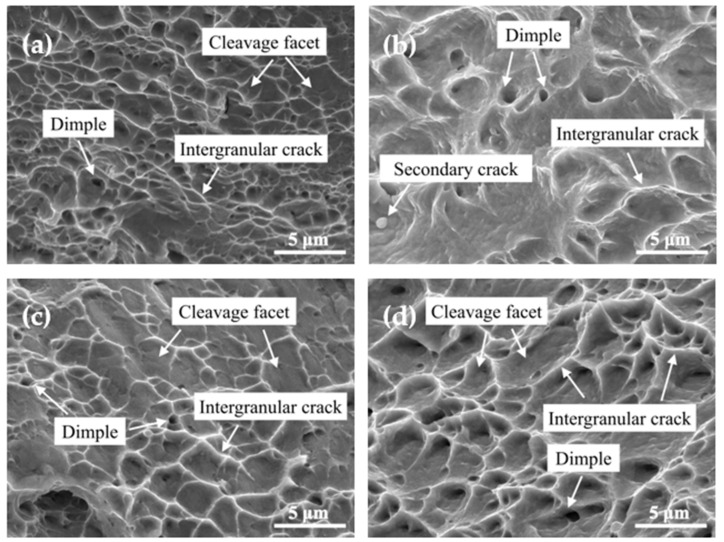
Tensile fracture morphologies of the alloys at different conditions. (**a**) C17200 in the unaged state; (**b**) QBe2.0 in the unaged state; (**c**) peak aging of C17200; (**d**) peak aging of QBe2.0.

Figure 2 shows the typical fracture morphologies of the tensile specimens under different aging conditions. The alloy fractures all show quasi-dissociative fracture morphologies, with a small number of along-crystal cracks, secondary cracks, and tough nest structures on the dissociative surface [16,17]. Compared with the initial state (unaged), the tough nests of C17200 were denser and deeper than those of QBe2.0, and the elongation is better. The size and depth of the tough nests have a significant effect on the plasticity of the alloy. In the initial state, the tough nests of the C17200 alloy were denser and deeper than those of QBe2.0, and therefore, its elongation was better. In addition, the secondary cracks in QBe2.0 can effectively prevent the movement of dislocations during the tensile process, thus improving the tensile strength of the alloy [18]. As the aging process proceeds, precipitated phases form in the alloys, which are usually harder than the matrix and will not deform unless they are broken or torn from the matrix. This leads to the development of cracks and facilitates the expansion of intergranular cracks. Immediately adjacent to the leading edge of the crack, a cavity similar to a plastic pit is formed as the stress state is essentially three-way, so the crack initiation mechanism comes into play. It can be seen that this type of fracture has the general morphology of a ductile fracture, as if the precipitated phase is not distributed within the grain but is concentrated at the grain boundaries, so that the crack can expand along the grain boundaries, which results in a degree of “plastic along grain” fracture [17]. Therefore, the fracture surfaces of the two alloys after aging show progressively shallower toughness nests and larger deconvolution surfaces, which further explains the gradual decreases in elongation.

### 3.2. Microstructure Observations

**Figure 3:**
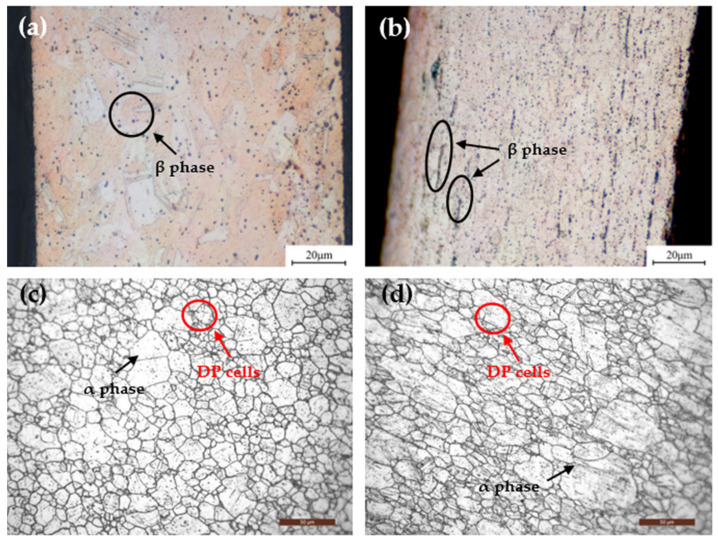
The longitudinal sections and surface microstructures of the alloys at the peak aging. (**a**,**c**) C17200 alloy; (**b**,**d**) QBe2.0 alloy.

Figure 3 illustrates the longitudinal cross-sectional and surface microstructures of the two alloys at peak aging. As shown in Figure 3a,b, a large number of dark particles appear in the alloys after aging, which are identified as β phases under subsequent microstructure observations [19]. The β phase of the C17200 alloy exhibits a dispersed point-like distribution, while the β phase of the QBe2.0 alloy presents a chain-like distribution, and the number density is greater than that of the C17200 alloy. In addition, the β phase in beryllium copper is relatively brittle, leading to stress concentrations distributed along a chain. It is easy to crack during the forming process, which means that the strength of QBe2.0 is much lower than that of the C17200 alloy.

The surface microstructures of the alloys at peak aging are presented in Figure 3c,d. After aging, it can be observed that the microstructures consist of the α phase (the solid solution phase) and a small number of discontinuous precipitation (DP) cells located at the grain boundaries (GBs) of a phase [20]. The crystal grains of the C17200 alloy are more finely and more uniformly distributed in the equiaxed shape, while the crystal grains in the QBe2.0 alloy are distributed in long strips in the matrix. The typical cold-rolled deformation characteristics that evolve into a lamellar structure along the rolling direction are retained in the peak aging alloys, as shown in Figure 3d. The grain size of the C17200 alloy was approximately 0.02 mm, while the grain size of the QBe2.0 alloy was 0.025 mm, and a few were 0.035 mm. Furthermore, DP cells involve the formation of a solute-depleted matrix phase (α’) and a precipitate phase (γ) as a duplex transformation product, and they usually nucleate at the GBs and grow into one side of the supersaturated matrix (the α phase) [21,22]. The DP cells consumed large amounts of solute atoms and contained a coarse equilibrium phase of γ instead of fine metastable γ’ phases, resulting in weakening the precipitation strengthening of the alloy [23,24]. Consequently, the strength of the alloy would decrease with the increase in the volume fraction of DP cells, which is consistent with the relatively lower strength of the QBe2.0 alloy than that of the C17200 alloy.

### 3.3. EBSD Analysis of the Microscopic Structure

**Figure 4:**
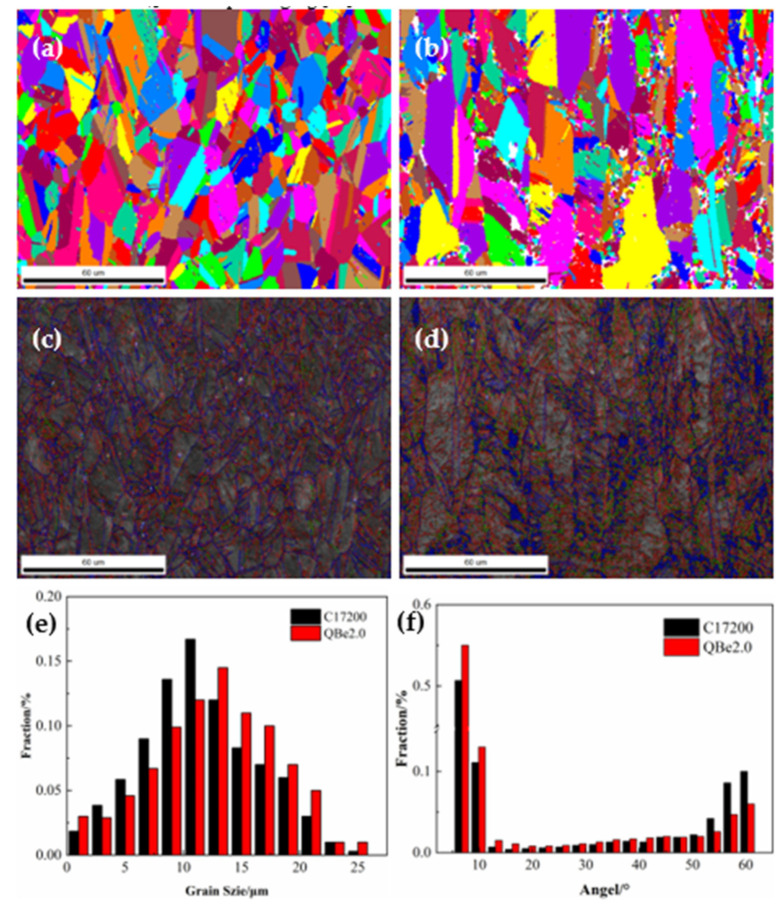
EBSD micrographs and orientation maps of alloys at the peak aging. (**a**,**c**) C17200; (**b**,**d**) QBe2.0; (**e**) grain size of the tested alloys; (**f**) misorientation angle distributions of the tested alloys. HAGBs are shown as blue lines, and LAGBs are shown as red lines.

The EBSD characteristics of the tested alloys at peak aging are presented in Figure 4. As shown in Figure 4a,b, the grains of C17200 are equiaxed and evenly distributed in the matrix, whereas the grain distribution of QBe2.0 is not uniform and exhibits a pronounced growth trend along the rolling direction. The uneven grain distribution of the QBe2.0 alloy causes incompatible plastic deformation and local stress concentrations [25]. Moreover, with a large number of fine grains positioned around the elongated grains, the process of recovery and recrystallization is accelerated by acting as nucleation points for recrystallization at high temperatures; thus, the strength of the alloy decreases significantly [26,27]. The grain sizes present standard normal distributions in Figure 4e, and the average grain sizes of the C17200 and QBe2.0 alloys reached 5 μm and 9 μm, respectively. Therefore, it can be concluded that the grain size and uniformity of the C17200 alloy are better than those of the QBe2.0 alloy, resulting in a higher grain strengthening effect.

The orientation maps of the two alloys at peak aging are presented in Figure 4c,d with high-angle grain boundaries (HAGBs) shown as blue lines and low-angle grain boundaries (LAGBs) shown as red lines. According to the statistical analysis of the misorientation angles shown in Figure 4f, grain orientations lower than 10° account for 21% and 17% of the grain boundaries in the C17200 and QBe2.0, respectively. In general, the number of small-angle grain boundaries (less than 10 °) is positively correlated with the dislocation density inside the alloy. The larger the number of small-angle grain boundaries is, the more significant the hindering effect on the movement of dislocations. Furthermore, the number density of twins of C17200 is twice that of QBe2.0, reaching 22% and 11%, respectively. Since slipping from the twin crystal requires more energy during the movement of the dislocation, the mechanical properties of the alloy improve by obstructing the slip and movement of the dislocation so that the strength of C17200 was higher than that of QBe2.0 at peak aging [28].

### 3.4. Microstructure Observations by TEM

**Figure 5:**
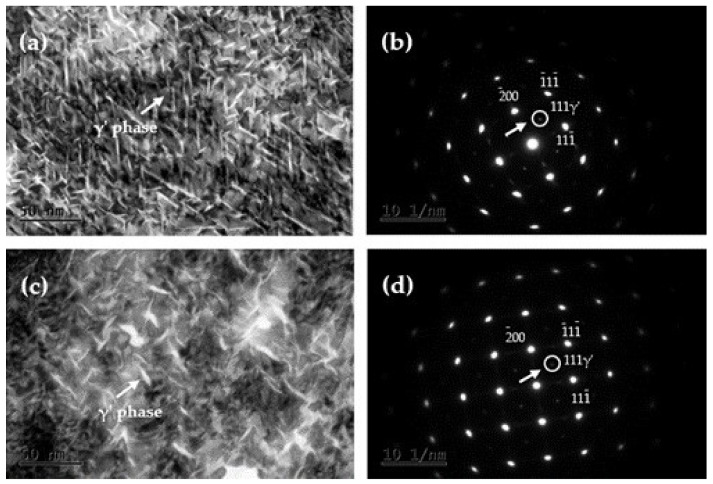
TEM images of alloys at the peak aging. (**a**) Bright field TEM image of C17200; (**b**) SAED corresponding to (**a**,**c**) bright field TEM image of QBe2.0; (**d**) SAED corresponding to (**c**).

Figure 5 illustrates the TEM images of the tested alloys at the peak aging. Cu-Be alloys are typical age-strengthened alloys, and the α phase is a solid solution of Be in the Cu matrix [29]. It has a face-centered cubic structure and good plasticity. From the TEM bright-field images, it can be seen that a large number of strip-like γ’ phases with lengths of 20–50 nm were dispersed in the matrix, as shown in Figure 5a,c. According to the corresponding SAED maps shown in Figure 5b,d, these elongated strip-like precipitates have obvious habitual planes parallel to the (113)_α_ and (112)_α_ crystal planes of the matrix. The γ’ phase is a metastable phase with a transition structure, with a body-centered tetragonal (BCT) structure, and its lattice constants are a = 0.254 nm and b = c = 0.279 nm [30]. Furthermore, the γ’ phase is in a semicoherent relationship with the matrix, resulting in a large lattice distortion that hinders the movement of dislocations. The observations show that the γ’ phase is the main strengthening phase of beryllium bronzes, and the mechanical properties of the Cu-Be alloys are mainly obtained by precipitating the γ’ phase from the supersaturated solid solution α phase. Therefore, it can be seen that the number density of the γ’ phase in the C17200 alloy is much larger than that seen in the QBe2.0 alloy, indicating that the precipitation is sufficient, which enhances the precipitation strengthening effect and improves the strength of the alloy.

**Figure 6:**
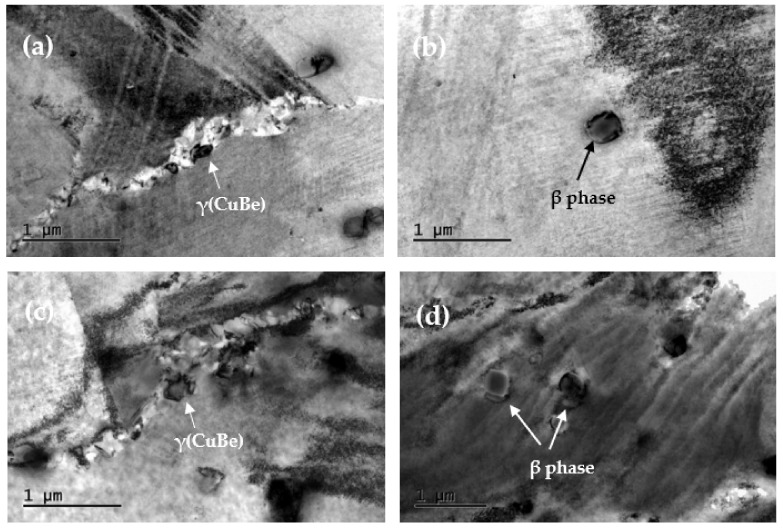
TEM images of alloys at the peak aging. (**a**) γ phase of C17200; (**b**) β phase of C17200; (**c**) γ phase of QBe2.0; (**d**) β phase of QBe2.0.

Further observation of the alloy precipitates by TEM found that, in addition to the large number of γ’ phases precipitated in the alloy matrix, there were also a small number of black particles distributed along the grain boundaries, as shown in Figure 6a,c. The particle phase is determined to be the equilibrium phase γ precipitated by beryllium and nickel in the alloy, and their size is 0.1–0.5 μm, which is much larger than the size of the metastable γ’ phase. During the aging of Cu-Be alloys, the precipitation phase always preferentially precipitates and grows at the grain boundary of the α phase, which leads to the inhomogeneous structure and properties of the alloy [31]. According to the research, the γ phase at the grain boundary is actually caused by discontinuous desolation (cellular desolation). This precipitation method is a two-phase process, α → α’ + γ, where α is a supersaturated solid solution and γ is an equilibrium phase [32,33,34]. The α’ phase and the α phase have the same crystal structure and are separated by an interface but have different compositions (by way of depletion of the solute elements). In discontinuous desolation, solute redistribution is carried out by interface diffusion rather than by matrix volume diffusion [35]. With increasing aging time, the γ phase formed by discontinuous desolation gradually aggregates and grows at the grain boundary and diffuses into the grain. As the cellular products diffuse, the coarse equilibrium phase dissolves the previously formed fine metastable phase γ’ deteriorating the mechanical properties of the material.

In addition, a large amount of micron-scale β phase material is present in both alloys, as shown in Figure 6b,d. The β phase is a disordered solid solution with a body-centered cubic structure that has the characteristics of high hardness and brittleness. According to the analysis of the phase diagrams, the β phase is stable only above 605 °C, and the transformation process of the β phase is very fast and can only be suppressed during solution treatment [36]. Comparing the two alloys, the size of the β phase in C17200 is 0.05–0.2 μm, while the size of the β phase in the QBe2.0 alloy is 0.5–0.8 μm, and its number is significantly larger than that of the C17200 alloy, which reduces the effect of precipitation strengthening. This is consistent with the previous microstructural observations of the alloys. At the same time, the large precipitates will act as the core of microcrack nucleation and reduce the plasticity of the material in the process of plastic deformation, thereby decreasing the elongation of the material with aging.

Above all, the differences in the mechanical properties of the C17200 alloy and the QBe2.0 alloy are mainly related to their microstructures, including grains, grain boundaries, twins, and precipitates. The grain structure of the C17200 alloy is mostly uniform, small and equiaxed, while the grain structure of the QBe2.0 alloy is different in size and elongated. At the same time, the grain size, uniformity, small angle grain boundary, and twin density of the C17200 alloy are all better than those of the QBe2.0 alloy, which leads to more significant effects of grain refinement, dislocation, and twin strengthening. In addition, the precipitation density of the γ’ strengthening phase in the C17200 alloy is much greater than that of the QBe2.0 alloy, the precipitation is more complete, and the precipitation strengthening effect is more obvious. Meanwhile, the morphologies and amounts of the γ phases in the alloys are almost the same, but the number and amounts of the brittle β phases are smaller than those of the QBe2.0 alloy. Therefore, under the combined effects of the various strengthening mechanisms, the C17200 alloy exhibits better mechanical properties.

## 4. Conclusions

In this paper, the mechanical properties of the C17200 alloy and the QBe2.0 alloy were investigated, and the microstructures of the alloys at peak aging (300 °C for 2 h) were analyzed. The conclusions were as follows:

(1) In the peak-aged state, the tensile and yield strengths of the C17200 alloy were much higher than those of QBe2.0 alloy. Compared with the initial state, the strength increment of the C17200 alloy after aging was significantly higher than that of the QBe2.0 alloy, and the elongation was significantly reduced.
(2) The grain size of C17200 alloy was smaller and the distribution was more uniform than those of QBe2.0 alloy. Moreover, the percentages of low-angle grain boundaries and twin density in C17200 alloy were much higher than those in QBe2.0 alloy, resulting in a more pronounced grain boundary strengthening effect.
(3) A large number of γ’, γ and β phases were precipitated in both alloys. The density of the γ’ phase in the C17200 alloy was greater, while the amounts of the brittle β phase were smaller than those of the QBe2.0 alloy.
(4) The higher strength of the C17200 alloy was mainly caused by the combined effects of grain refinement strengthening, dislocation strengthening, twin strengthening, and precipitation strengthening.

## Figures and Tables

**Table 1:** The chemical compositions of the tested alloys, wt.%.

Alloy	Ni	Co	Be	Al	Fe	Si
C17200 (Standard)	≥0.2		1.8–2.0	≤0.2	Fe + Ni + Co ≤ 0.6	≤0.2
C17200 (Tested)	<0.01	0.22	2.04	0.034	0.038	0.056
QBe2.0 (Standard)	0.2–0.5	/	1.8–2.1	≤0.15	≤0.15	≤0.15
QBe2.0 (Tested)	0.23	0.042	1.87	0.044	0.066	0.064

## Data Availability

The data presented in this study are available on request from the corresponding author.
